# Biosynthesis and Metabolic Fate of Phenylalanine in Conifers

**DOI:** 10.3389/fpls.2016.01030

**Published:** 2016-07-13

**Authors:** María B. Pascual, Jorge El-Azaz, Fernando N. de la Torre, Rafael A. Cañas, Concepción Avila, Francisco M. Cánovas

**Affiliations:** Departamento de Biología Molecular y Bioquímica, Facultad de Ciencias, Universidad de MálagaMálaga, Spain

**Keywords:** trees, aromatic amino acids, phenylpropanoids, nitrogen recycling, gene regulatory networks

## Abstract

The amino acid phenylalanine (Phe) is a critical metabolic node that plays an essential role in the interconnection between primary and secondary metabolism in plants. Phe is used as a protein building block but it is also as a precursor for numerous plant compounds that are crucial for plant reproduction, growth, development, and defense against different types of stresses. The metabolism of Phe plays a central role in the channeling of carbon from photosynthesis to the biosynthesis of phenylpropanoids. The study of this metabolic pathway is particularly relevant in trees, which divert large amounts of carbon into the biosynthesis of Phe-derived compounds, particularly lignin, an important constituent of wood. The trunks of trees are metabolic sinks that consume a considerable percentage of carbon and energy from photosynthesis, and carbon is finally immobilized in wood. This paper reviews recent advances in the biosynthesis and metabolic utilization of Phe in conifer trees. Two alternative routes have been identified: the ancient phenylpyruvate pathway that is present in microorganisms, and the arogenate pathway that possibly evolved later during plant evolution. Additionally, an efficient nitrogen recycling mechanism is required to maintain sustained growth during xylem formation. The relevance of phenylalanine metabolic pathways in wood formation, the biotic interactions, and ultraviolet protection is discussed. The genetic manipulation and transcriptional regulation of the pathways are also outlined.

## Introduction

**FIGURE 1:**
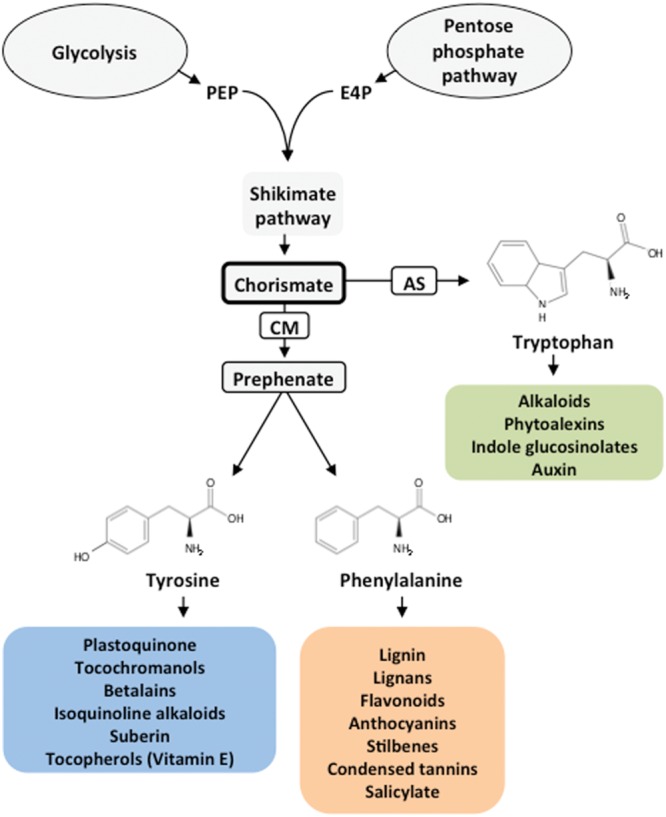
**General pathway for aromatic amino acid biosynthesis and derived products.** The shikimate pathway connects central carbon metabolism (glycolysis and pentose phosphate pathways) with the biosynthesis of aromatic amino acids and derived products. PEP, phosphoenolpyruvate; E4P, erythrose 4-phosphate; CM, chorismate mutase; AS, anthranilate synthase.

Animals, including humans, are unable to synthesize all the amino acids required for protein synthesis and metabolic nitrogen (N) homeostasis. These amino acids are termed essential and should be supplied in the diet direct or indirectly from a plant source. Essential amino acids are synthesized in the chloroplasts of photosynthetic organs and other non-green plastids of plants. The capacity of plants to synthesize a set of essential amino acids may be linked to the acquisition of a set of prokaryotic genes that are responsible for this function during the primary endosymbiosis event that resulted in the plastids (de la Torre et al., 2014). These findings may explain the inability of humans and many animals to synthesize essential amino acids and why they should be provided in their foods and feeds. One such dietary amino acid, phenylalanine (Phe), is a critical metabolic node that plays an essential role in the interconnection between the primary and secondary metabolism of plants. Phe is a protein building block and a precursor of numerous compounds that are crucial for plant reproduction, growth, development and defense against different types of stresses. Phe-derived compounds include phenylpropanoids, flavonoids, anthocyanins, lignin, lignans, stilbenes, condensed tannins and the important plant hormone salicylate (**Figure 1**).

The metabolism of Phe plays a central role in the channeling of carbon from photosynthesis to the biosynthesis of phenylpropanoids. The study of Phe metabolism is particularly relevant in trees, such as conifers, that divert large amounts of carbon into the biosynthesis of these Phe-derived compounds, particularly lignin, an important constituent of wood. It is estimated that nearly 30–40% of photosynthetically fixed carbon is channeled through phenylalanine for the biosynthesis of lignin during wood formation. Consequently, the trunks of trees are powerful metabolic sinks of the fixed carbon in photosynthetic tissues that is immobilized in wood (Craven-Bartle et al., 2013). Conifers also exhibit a highly complex secondary metabolism derived from aromatic amino acids, and therefore these trees are interesting models for studying these pathways (Warren et al., 2015). Conifers are the most abundant group of extant gymnosperms and dominate large ecosystems in the northern hemisphere that make an important contribution to global carbon fixation. Coniferous trees are also of great economic importance because they are the primary source for timber and paper production worldwide (Farjon, 2010). Many comprehensive reviews of the biosynthesis of aromatic amino acids in plants were published recently (Tzin and Galili, 2010; Maeda and Dudareva, 2012). However, less attention has been paid to the importance of Phe metabolism in woody plants. This review presents the biosynthesis and principal metabolic fates of Phe in conifers.

## Biosynthesis of Phenylalanine, Deamination, and Nitrogen Recycling

**FIGURE 2:**
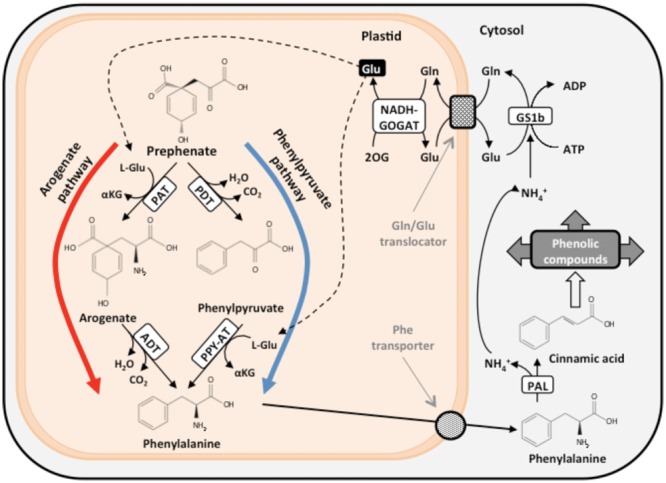
**The two alternative pathways for Phe biosynthesis and associated N recycling.** PAT, prephenate aminotransferase; PDT, prephenate dehydratase; ADT, arogenate dehydratase; PPY-AT, phenylpyruvate aminotransferase; PAL, phenylalanine ammonia lyase; GS1b, glutamine synthetase 1b; NADH-GOGAT, NADH-dependent glutamate synthase; L-Glu, L-glutamate; L-Gln, L-glutamine; 2-OG, 2-oxoglutarate.

The carbon skeletons required for the biosynthesis of aromatic amino acids are channeled from photosynthesis through the shikimate pathway toward the biosynthesis of chorismate, a common precursor for the synthesis of Phe, Tyr, and Trp (Maeda and Dudareva, 2012). Chorismate may be used by the enzyme anthranilate synthase in the synthesis of Trp or converted to prephenate, which is the direct precursor for the biosynthesis of Tyr and Phe. Two alternative routes for the Phe biosynthesis have been identified (**Figure 2**). Prephenate is transaminated by prephenate-aminotransferase (PAT) to generate arogenate in the arogenate pathway (Maeda and Dudareva, 2012). In a next step, arogenate is decarboxylated and dehydrated by the catalytic action of arogenate dehydratase (ADT) to yield Phe (Bonner and Jensen, 1987a). Alternatively, the decarboxylation and dehydratation of prephenate yields phenylpyruvate in a reaction catalyzed by the enzyme prephenate dehydratase (PDT) through the phenylpyruvate pathway. Subsequently, phenylpyruvate is transaminated to Phe by a phenylpyruvate-aminotransferase (Fischer and Jensen, 1987a). The phenylpyruvate pathway is utilized for Phe biosynthesis in microorganisms, and the arogenate pathway has been proposed to predominate in the biosynthesis of Phe in plants (Maeda et al., 2010). However, the functionality of these pathways was analyzed in only in a small number of angiosperm species. Prephenate and arogenate are also precursors for the biosynthesis of Tyr via two alternative pathways mediated by prephenate dehydrogenase and arogenate dehydrogenase, respectively (Bonner and Jensen, 1987b; Fischer and Jensen, 1987b). Conifers, unlike angiosperms, possess a plastid-located folate-dependent phenylalanine hydroxylase that catalyzes the interconversion between Phe and Tyr to provide enhanced plasticity to the aromatic metabolism network (Pribat et al., 2010). Finally, Phe is transported outside the plastid to the cytosol and converted into trans-cinnamic acid, which is the first metabolite in the phenylpropanoid pathway, with the concomitant release of an ammonium molecule in the reaction catalyzed by phenylalanine ammonia-lyase (PAL).

An efficient N recycling mechanism is required to maintain the metabolic flux through the pathway and re-assimilate the released ammonium into glutamate and reincorporate it back into the Phe biosynthetic pathway by prephenate and phenylpyruvate aminotransferases (**Figure 2**; Cánovas et al., 2007; Craven-Bartle et al., 2013). Otherwise, plants undergoing active Phe biosynthesis would require a continuous input of external N to avoid severe N deficiency. This N recycling is particularly important in trees to maintain sustained growth in height during xylem differentiation and wood formation. Phe biosynthesis in the vascular cells of conifers is located in the plastid, and Phe deamination occurs in the cytosol. N recycling involves enzymes in the cytosol (GS1b) and plastid (NADH-GOGAT). Therefore, the actions of a glutamine translocator and a phenylalanine transporter are necessary to provide Phe for a variety of metabolic pathways, including lignin biosynthesis during wood formation. A glutamine/glutamate translocator was characterized in maritime pine cells that may be involved in glutamine import into the chloroplast and glutamate export to the cytosol, which prevents N loss from this essential pathway (Claros et al., 2010). The activity of a phenylalanine transporter in the plastid membrane has not been demonstrated, and the molecular and kinetic characteristics of this amino acid transporter are not known. More knowledge of the regulation of Phe transport during xylem development and in response to a variety of environmental stresses is required to obtain a more complete view of Phe metabolism.

The existence in conifers of a very diverse set of Phe-demanding pathways makes these plants an attractive candidate for a thorough analysis to clarify the routes of biosynthesis of this important amino acid. A multigene family encodes the ADT enzyme in conifers, and this family includes more members in gymnosperms than angiosperms (El-Azaz et al., 2016). ADT genes in maritime pine are differentially expressed, which suggests that these genes play different biological roles. N-terminal plastid-targeting sequences were predicted for all maritime pine ADT enzymes and a plastid location was also experimentally confirmed for many of these enzymes. Some of these ADT enzymes also exhibited PDT activity, which supports these enzymes as potential candidates in the alternative phenylpyruvate pathway for Phe biosynthesis. El-Azaz et al. (2016) identified a 22-amino acid region in the C-terminal of these enzymes as being responsible for conferring PDT activity (PAC-domain). This small protein domain is of prokaryotic origin and it has been shown to be present in all plant clades. Phylogenetic analyses suggest that various subfamilies of ADTs evolved from an ancestral PDT/ADT subfamily that existed in the most ancient plant clades (El-Azaz et al., 2016). Despite this functional diversification, the ancient pathway using phenylpyruvate as intermediary has been preserved throughout the evolution of plants for Phe biosynthesis. The availability of a diverse catalog of enzymes for the biosynthesis of Phe has likely allowed a more specialized ability to cope with multiple developmental and physiological situations that demand the biosynthesis of Phe-derived compounds.

Taken together, the above results suggest that conifers may possess a greater and specialized flexibility for the biosynthesis of Phe that facilitates adaptation to different physiological and environmental conditions. Consistent with the above findings, gymnosperms, compared to angiosperms, exhibit a more phylogenetically diverse set of PAL enzymes that catalyze the first and committed step in the phenylpropanoid pathway (Bagal et al., 2012). The recent release of several conifer genomes will aid in the identification of other putative specificities in the biosynthesis of Phe in these plants and reveal the conservation of these pathways throughout the evolution of land plants.

## Phe-Derived Compounds/Phenylpropanoids

Phenylpropanoids are an important group of phenolic plant secondary metabolites derived from Phe although in certain plants can be also derived from Tyr. Phenylpropanoids are compounds that contain a phenyl ring with a C3 side chain (Heldt and Piechulla, 2011). This structure is conserved only in monolignols and their metabolic intermediaries, but the term “phenylpropanoids” also include metabolites derived from these compounds and intermediaries of Phe synthesis, such as flavonoids, stilbenes, coumarins, salicylate and gallate, which exhibit very divergent structures. Phenylpropanoids and derivatives can polymerize producing compounds such as lignans, lignin, condensed and hydrolysable tannins, and may be part of suberin and cutin (Heldt and Piechulla, 2011). All of these compounds have important biological activities and serve as cell wall constituents, antibiotics, light protectants and flower pigments and participate in the formation of impermeable layers.

## Phenylpropanoids Involved in Wood Formation in Conifers

The monolignol synthesis pathway has received much attention because these compounds are the precursors of lignin, which is the most abundant organic compound on Earth, after cellulose. Lignin comprises 30% of the plant biomass, and it is an essential component of wood (Boerjan et al., 2003). Therefore, the synthesis of these metabolites is as powerful sink that uses a considerable percentage of the carbon and energy derived from photosynthesis. Lignin composition and characteristics possess a huge economic importance because they determine wood properties (Boerjan et al., 2003).

**FIGURE 3:**
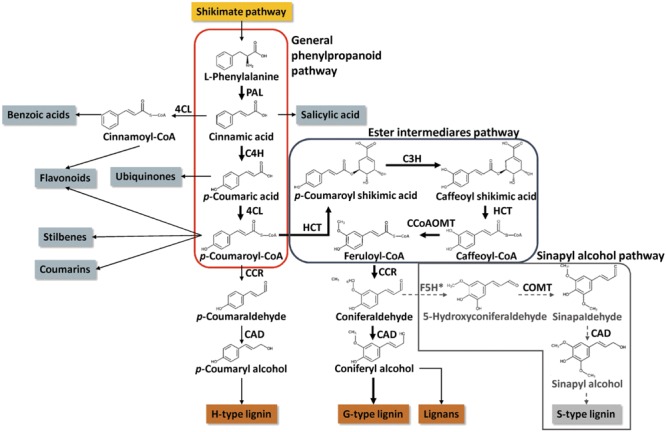
**Simplified scheme of monolignol synthesis pathway in conifers.** Thick arrows highlight the route channeling the higher amount of carbon in conifers, the synthesis of coniferyl alcohol. The gray and discontinued arrows highlight the sinapyl alcohol synthesis that does occur in conifers because of the lack a gene encoding the F5H enzyme. Asterisk highlights the lack of F5H enzyme in conifers. Phenylalanine ammonia-lyase (PAL), cinnamate 4-hydroxylase (C4H), 4-coumarate:CoA ligase (4CL), cinnamoyl-CoA reductase (CCR), cinnamyl-alcohol dehydrogenase (CAD), shikimate *O*-hydroxycinnamoyltransferase (HCT), *p*-coumarate 3-hydroxylase (C3H), caffeoyl-CoA *O*-methyltransferase (CCoAOMT), ferulic acid 5-hydroxylase/coniferaldehyde 5-hydroxylase (F5H), and caffeic acid 3-*O*-methyltransferase (COMT).

The metabolic pathway leading to the biosynthesis of lignin was extensively studied in several plant species, including conifers. In contrast, other topics, such as its demonstrated plasticity (Cass et al., 2015) or the precise transcriptional network that regulates these pathways, are much less studied. However, these topics are fundamental to our understanding of the multiple mechanisms that are critical for plant development and environmental adaptation. Conifers possess enzymes similar to those of angiosperms for the synthesis of *p*-coumaryl M1H and coniferyl M1G monolignols but lack the enzymatic machinery needed to synthesize sinapyl M1S subunits (**Figure 3**; see Wagner et al., 2012 for a comprehensive description of the biosynthesis of monolignols in conifers). Recent publications of the genome sequences of *Picea glauca* (Birol et al., 2013; Warren et al., 2015), *P. abies* (Nystedt et al., 2013) and *Pinus taeda* (Neale et al., 2014; Zimin et al., 2014) enabled the identification of full-length gene models for all enzymes in the monolignol biosynthetic pathway, except ferulic acid 5-hydroxylase/coniferaldehyde 5-hydroxylase (F5H). This result is consistent with the lack of F5H activity in conifers, which prevents the existence of S units in the lignin of these species (Wagner et al., 2015). The lack in S units yields a more condensed lignin, which is more difficult to remove from the polysaccharide components of the cell wall matrix during industrial processing (Ralph, 2010). This is an important issue because conifer forests dominate large extensions of the Northern hemisphere and provide approximately the 60% of the wood used for industrial purposes (Farjon, 2010). Therefore, the elucidation and manipulation of the phenylpropanoid pathway in conifers is of great interest.

## Investigation of Phenylpropanoid Metabolism Using Genetic Manipulation in Conifers

The discovery of mutants and the generation of transgenic lines in conifers is extremely difficult compared to herbaceous plants, such as *Arabidopsis*, but monolignol synthesis and lignification in conifers was investigated using transgenic lines and transformed cell cultures induced to differentiate tracheary elements. (Möller et al., 2003, 2005; Wagner et al., 2005, 2007, 2009, 2011, 2013, 2015; Wadenbäck et al., 2008; Bedon et al., 2009; Trontin et al., 2014).

The first enzyme in the synthesis of monolignols, 4-coumarate:CoA ligase (4CL), was investigated using genetic manipulation (Wagner et al., 2009). The activity of 4CL is essential for secondary metabolism because it is responsible for *p*-coumaroyl-CoA production, which is the precursor of several types of secondary metabolites, such as monolignols, flavonoids, stilbenes, and coumarins (**Figure 3**) (Cheynier et al., 2013). The role of 4CL was examined using the generation of RNAi transgenic lines in *P. radiata* (Wagner et al., 2009). Suppression of 4CL gene expression exerted the biggest impact on lignin production of all of the genetic manipulations of phenylpropanoid related genes in conifers (Wagner et al., 2012). The transgenic plants exhibited a reduced lignin content of approximately 50%. This enzyme acts at the beginning of the metabolic pathway, but the accumulation of certain levels of lignin is explained by the existence of a small 4CL gene family in conifers (Wei and Wang, 2004; Koutaniemi et al., 2007). Notably, the decrease in lignin content in the 4CL RNAi lines was accompanied by an increase in flavonoids and bark (tissue rich in tannins, a flavonoid polymer) and alterations in carbohydrate metabolism (Wagner et al., 2009). These findings support different roles for each 4CL paralog and, possibly, the ability to redistribute the C surplus caused by a decrease in monolignol synthesis. Therefore, gene co-expression analyses in conifers highlighted the existence of co-regulatory mechanisms between different pathways of secondary metabolism (e.g., synthesis of Phe, monolignols, flavonoids, and terpenoids), which are likely mediated by Myb transcription factors (Cañas et al., 2015; Raherison et al., 2015). The formation of membrane-associated enzyme complexes for the channeling and regulation of metabolite synthesis was suggested (Mast et al., 2010). This may explain why another member of the gene family does not completely replace the role of the 4CL gene after suppression.

Other enzymes that were investigated using genetic manipulation include shikimate *O*-hydroxycinnamoyltransferase (HCT), caffeoyl-CoA *O*-methyltransferase (CCoAOMT), the cinnamoyl-CoA reductase (CCR; Wagner et al., 2007, 2011, 2013; Wadenbäck et al., 2008) and the most widely studied, cinnamyl-alcohol dehydrogenase (CAD; Möller et al., 2003, 2005; Wagner et al., 2005; Bedon et al., 2009; Trontin et al., 2014). Suppression of gene expression generally decreased lignin content, and this decrease was more pronounced when the modifications included enzymes that are involved in the first steps of the pathway. This was accompanied by alterations in the metabolic profiles and composition of lignin. For example, these metabolic changes in the stems of *P. pinaster* CAD RNAi lines affected C metabolism and N metabolism (Trontin et al., 2014). Taken together, the data obtained from all of these cell lines support a metabolic plasticity in lignin biosynthesis and the incorporation of non-traditional compounds to lignin (Wagner et al., 2012). This flexibility may be explained by the existence of gene families that code the enzymes of the phenylpropanoid metabolism and their promiscuity in substrate use.

**FIGURE 4:**
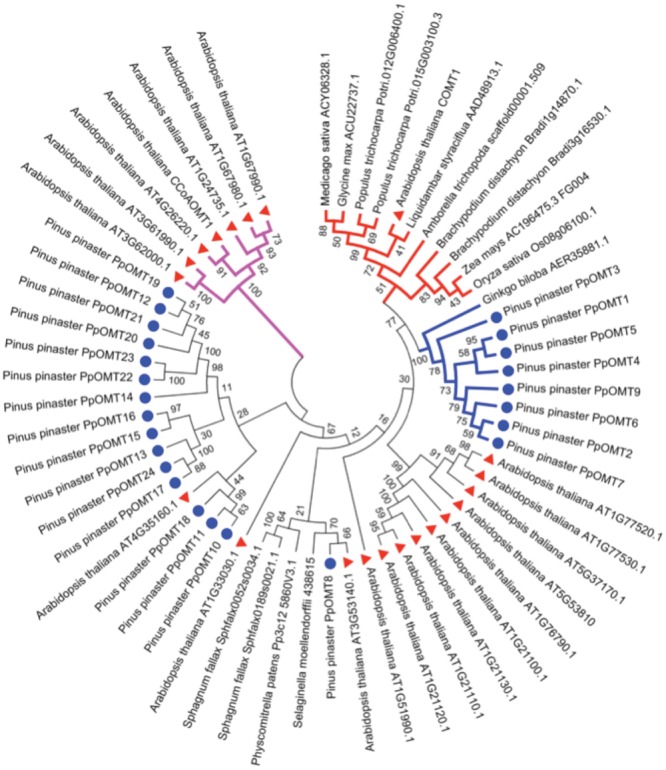
**Phylogenetic tree of the deduced protein sequences of plant genes encoding caffeic acid 3-*O*-methyltransferase (COMT).** In the tree, the protein sequences correspond to virtually all the sequences of *O*-methyltransferases from *Arabidopsis thaliana*, all the sequences of *O*-methyltransferases, type COMT, found in the *Pinus pinaster* SustainPineDB and the most similar sequence to Ath-COMT1 from the rest of the shown species. The CLUSTALW program was used for sequence alignments (Thompson et al., 1994). The evolutionary history was inferred using the Neighbor-Joining method (Saitou and Nei, 1987) with 1000 bootstrap replications. The optimal tree with the sum of branch length = 12.56422456 is shown. The evolutionary distances were computed using the Poisson correction method (Zuckerkandl and Pauling, 1965) and are in the units of the number of amino acid substitutions per site. The analysis involved 60 amino acid sequences. All positions containing gaps and missing data were eliminated. There were a total of 156 positions in the final dataset. Evolutionary analyses were conducted in MEGA6 (Tamura et al., 2013). The sequences used for the alignments and phylogenetic trees were obtained in Phytozome database (http://phytozome.jgi.doe.gov) and GenBank and, for *P. pinaster*, in SustainPineDB (http://www.scbi.uma.es/sustainpinedb/). The alignment, tree and accession numbers are available in TreeBase (http://purl.org/phylo/treebase/phylows/study/TB2:S18814). Red triangles correspond to *A. thaliana* sequences and the blue circles to *P. pinaster* sequences. Purple branches correspond to the Ath-CCoAOMT group of sequences, red branches to the angiosperm COMT group and blue branches to the gymnosperm COMT-like group.

The production of syringyl lignin was achieved recently in conifers using bioengineering approaches (Wagner et al., 2015). The authors of this work manipulated conifer cells for the first time to obtain tracheary elements that synthesized and incorporated sinapyl M1S alcohols into lignin. The synthesis of M1S alcohols in conifers is prevented by the lack of a gene encoding the ferulate 5-hydroxylase/coniferaldehyde 5-hydroxylase (F5H or CAld5H) enzyme. This enzyme transfers a hydroxyl group to the phenolic ring of the coniferaldehyde to produce 5-hydroxylconiferaldehyde, which is methylated by caffeic acid 3-*O*-methyltransferase (COMT) to synthesize sinapaldehyde, which is finally reduced by CAD to generate the sinapyl alcohol (M1S). These authors generated *P. radiata* tracheary elements transformed with F5H or F5H/COMT genes from the angiosperm plant *Liquidambar styraciflua* and produced lignin with S-type units. Notably, some authors considered that conifers lack genes encoding COMT enzymes, but the tracheary elements transformed with an angiosperm F5H gene produced synapyl alcohol at a detectable level (Wagner et al., 2015). This result suggests the existence of COMT activity in conifers, but with lower catalytic capacity, which was shown in the same work. Co-transformation with F5H and COMT resulted in huge amounts of synapyl alcohols compared to single transformants. The lack of the F5H gene may induce a different selection pressure on the *O*-methyltransferases, which previously exhibited COMT activity, and alter the affinity for substrates or the preferential use of new substrates such as flavonoids. A phylogenetic analysis of conifer *O*-methyltransferases shows a group of enzymes that are highly related to the COMT of angiosperms but with an amino acid identity near 55% (**Figure 4**).

Genetic manipulation studies in conifers remain limited despite the above–mentioned reports, and obtaining mutants is extremely complicated because of the long life cycles of conifer species. Only one mutant for a gene in monolignol metabolism was isolated and characterized, *cad-n1* in *P. taeda* (MacKay et al., 1997). However, the implementation of new genome editing techniques may greatly impact conifer biotechnology. Genome editing in model plant species has undergone extraordinary development in the last few years because of CRISPR/Cas9 technology (Schaeffer and Nakata, 2015). The specificity of this technique for the selection of genome regions to be edited permits the isolation of mutants for specific conifer genes and the substitution of genes with different functions. Mutant lines for determined phenotypes may be generated with truncated or eliminated genes using this technology. Genome editing of the 4-coumarate:CoA ligase (4CL) gene family was performed in *Populus* recently, which extended the use of this powerful CRISPR/Cas9 technology from model plant species to woody perennials (Tsai and Xue, 2015).

## Phenylpropanoids Involved in Biotic Interactions in Conifers

Most gymnosperms have long generation times, and some species are among the longest living plant species. These trees must cope with a wide range of stresses during their long life cycles, particularly different types of biotic stresses, such as fungi, insects, or herbivorous animals, which generate significant forest damage annually. The bark of gymnosperms functions as the first line of defense to delay or stop the establishment of pathogens. These plants also evolved complex chemical defense mechanisms that are associated with the production of different secondary metabolites including multiple terpenoids and phenolic metabolites (Warren et al., 2015).

Conifers possess a set of constitutive defenses against a range of potential attacking organisms. Phenylpropanoids play an essential role in these processes at different levels: (i) deposition of lignin and suberin on tissues enhances resistance to the penetration of small organisms; and (ii) chemical defenses, including phenolics, terpenoids, and alkaloids, are released throughout bark tissues and function as antifeedants and toxins. The cortical parenchyma of conifers contains large amounts of phenolics. Similarly, polyphenolic parenchyma cells are specialized for the synthesis and accumulation of phenolic compounds (Hudgins and Franceschi, 2004) with antifeedant and antifungal activities (Beckman, 2000). These cells store starch and lipids (Krekling et al., 2000), which makes them attractive bait for pathogens. Lignification of the secondary wall of the sclerenchyma cells can also functions as a mechanical defense against bark beetles. The formation of callus tissue in response to wounding also relies on the production of phenylpropanoids because this tissue becomes lignified, suberized and impregnated with phenolic compounds (Franceschi et al., 2005). Hammerbacher et al. (2014) recently demonstrated that other Phe-derived compounds, proanthocyanidins and flavanols, are produced by *P. abies* in response to fungal infection, and these compounds must be considered chemical defense compounds. Pathogen-induced early lignification of fibers also functions as a structural defense in *Pinaceae* (Hudgins et al., 2003).

The activation of inducible chemical defenses in conifers involves a very efficient broad range system that is highly dependent on the production of phenolics and terpenoids. Different studies demonstrated a correlation among wound- and pathogen-induced damage, polyphenolic parenchyma cell expansion and the accumulation of phenolic compounds (Franceschi et al., 2000; Kusumoto and Suzuki, 2003). This accumulation was also accompanied by a variation in the type of phenolics (Brignolas et al., 1995; Lieutier et al., 1996), which suggests that more specific and toxic compounds for pathogens are produced. These phenolics changes were accompanied by the transcriptional activation of the flavonoid and stilbene pathways (Brignolas et al., 1995). Krekling et al. (2000) demonstrated that seasonal changes also influenced the phenolic content within these cells. Similarly, Danielsson et al. (2011) reported a correlation between the *P. abies* resistance to the fungus *Heterobasidion annosum*, the production of piceasides and flavonoids and the induction of genes in the flavonoid and proanthocyanidin pathways. The existence of other flavonoids that confer resistance against other fungal pathogens in conifers was also described (Song et al., 2011; Oliva et al., 2015).

A complete understanding of conifers defenses is essential to manage future pests that may affect forests. The roles of different types of Phe-derived compounds in the defense of these plants is becoming more clearly defined, but it still requires of a deeper analysis of the metabolic pathways involved and the technical capacity to characterize and identify these compounds. The recent characterization of various conifer genomes is a fundamental and promising resource (Birol et al., 2013; Nystedt et al., 2013; Neale et al., 2014; Warren et al., 2015).

## Phenylpropanoids Involved in Ultraviolet Protection in Conifers

Solar ultraviolet radiation potentially generates multiple negative effects in plants, such as damage to proteins, lipids, or DNA; growth reduction; or the inhibition of photosynthesis (Jordan, 2002; Frohnmeyer and Staiger, 2003; Barnes et al., 2016). Plants have developed various protection mechanisms to face these threats, including the biosynthesis of flavonoids and related phenylpropanoids that function as “UV sunscreens” and antioxidants (Caldwell, 1971; Yamaguchi et al., 2009; Agati et al., 2013). These compounds accumulate in vacuoles of the subepidermal cells of leaves protecting the inner cell layers from UV-B damage (Weissenböck et al., 1986; Landry et al., 1995; Kaffarnik et al., 2006). The presence of hydroxycinnamic acids and flavonol glycosides conjugated to the cell walls of multiple conifer species was also demonstrated (Strack et al., 1988).

Conifers are capable of growing in very different latitudes and heights, and these trees tolerate extremely different doses of UV radiation. The needles of these plants are rich in UV-B-absorbing soluble and cell wall-bounded phenolic compounds, such as lignans, coumarins, flavonoids, stilbenes, and hydroxycinnamic acids (Strack et al., 1988; Schnitzler et al., 1996; Fischbach et al., 1999). Notably, these conifer needles absorb short wavelength radiation, unlike herbaceous plants (DeLucia et al., 1992). In *P. sylvestris* and *P. abies*, the flavonol 3-*O*-glycoside acylated by with p-coumaric acid at position 3″ and p-coumaric or ferulic acid at position 6″ of the molecule were described as the main UV-B screening pigments (Schnitzler et al., 1996; Fischbach et al., 1999). These compounds are synthesized following UV-B irradiation and accumulate in epidermal cells, where they exhibit their effective protective function (Schnitzler et al., 1996; Kaffarnik et al., 2006). The flavonol glycosides kaempferol, isorhamnetin, and quercetin were also detected in epidermal tissue isolated from *P. sylvestris* needles. Remarkably, the biosynthesis of these glycosides directly depends on the activity of two critical enzymes in the synthesis of flavonoids and phenylpropanoids, chalcone synthase and PAL (Schnitzler et al., 1996), which demonstrate their *de novo* synthesis.

Identification of the exact phenylpropanoids that are involved in each developmental stage or physiological scenario in conifers is highly important for the future biochemical engineering of these plants toward the generation of new and enhanced forest-derived products.

## Transcriptional Regulation of Gene Expression

Regulation of the biosynthesis and utilization of Phe is a complex process that involves the coordinated expression of genes encoding enzymes located in different subcellular compartments and cellular types. An understanding of the transcription regulatory network associated with phenylpropanoid and lignin biosynthesis in conifers is crucial for future applications for tree improvement and sustainable forest management.

Transcriptome analyses indicated that several genes coding for TFs are preferentially expressed during wood formation in plants (Demura and Fukuda, 2007; Du and Groover, 2010). The best characterized are a set of transcription factors in the MYB and NAC families, which control lignin biosynthesis during wood formation.

The subfamily of R2R3-Myb factors is one of the largest transcription factor families in plants, with an estimated number of greater than 100 members in each species (Martin and Paz-Ares, 1997). These TFs bind AC elements in the promoter regions of phenylpropanoid and lignin biosynthesis genes to activate transcription. In trees, EgMYB2 from *Eucalyptus grandis* and PtMYB1 and PtMYB4 from *P. taeda* bind AC elements in the promoter of their target genes, which are expressed in developing wood and cause secondary wall thickening. Constitutive overexpression of these MYB transcription factors in tobacco (EgMYB2 and PtMYB4), *Arabidopsis* (PtMYB4) or white spruce (PtMYB1 and PtMYB8) increased secondary-wall thickening or led to ectopic lignin deposition (Patzlaff et al., 2003a,b; Goicoechea et al., 2005; Bomal et al., 2008).

Phenylalanine metabolism is finely regulated in conifers, primarily through transcriptional control. High transcript levels for three key genes *GS1b, PAL*, and *PAT* were detected in the stem and roots of young trees, and the compression wood of adult maritime pine trees (Craven-Bartle et al., 2013). These genes contain AC elements in their promoter regions, and the consensus sequence CCAACCAC/A functions as a *cis*-regulatory element involved in the transcriptional activation that is mediated by the MYB8 transcription factor (Craven-Bartle et al., 2013). Phe is the precursor for phenylpropanoids, and the regulation of Phe biosynthesis and metabolic utilization should occur in a coordinate manner, possibly in the same cell types. The overexpression of *PtMYB8* in white spruce clearly revealed the involvement of this transcription factor in secondary cell wall biogenesis and in lignin deposition during compression wood formation (Bomal et al., 2008). The *Myb8* gene exhibited strong similarity with *AtMYB61*, which is expressed in xylem tissues of *Arabidopsis*, and it plays an important role in the regulation of lignification (Newman et al., 2004). The co-expression of *PAT, PAL, GS1b*, and *MYB8* transcripts in the same cellular types further supports a coordinated transcriptional regulation of the pathway. The accumulation of MYB8 transcripts in compression wood of white spruce and maritime pine (Bedon et al., 2007; Craven-Bartle et al., 2013) is consistent with the ectopic lignification that resulted from the constitutive overexpression of *AtMYB61* in *Arabidopsis* (Newman et al., 2004). Other transcription factors in other families, such as NtLIM1 and ACBF, also bind to AC elements. Antisense inhibition of *NtLIM1* expression in transgenic tobacco plants reduced the expression levels of several phenylpropanoid genes, including *PAL* and *CAD*, and the total lignin content of stems, which indicated that it was required for normal lignin biosynthesis (Kawaoka et al., 2000). The relationship between the ACBF protein and its possible involvement in lignin biosynthesis has not been established (Seguin et al., 1997).

Other well-characterized TFs that are involved in the regulation of wood formation belong to the NAC family. These proteins are plant-specific proteins with a highly conserved N-terminal NAC domain, which has been implicated in nuclear localization, DNA binding, and homo- and/or heterodimer formation with other NAC domain proteins (Olsen et al., 2005). Similar to the MYB family, the NAC transcription factors comprise a large gene family with more than 100 members in *Arabidopsis thaliana* (Ooka et al., 2003), *Oryza sativa* (Ooka et al., 2003; Nuruzzaman et al., 2010), *Glycine max* (Le et al., 2011), *Populus trichocarpa* (Hu et al., 2010), and *E. grandis* (Hussey et al., 2015). At least 37 genes encoding NAC proteins were identified in the *P. pinaster* genome (Pascual et al., 2015). The NAC proteins participate in many developmental processes, including secondary cell wall formation (Yamaguchi and Demura, 2010). A group of poplar wood-associated NAC domain proteins (PtrWNDs; Zhong and Ye, 2007; Zhong et al., 2010b) and an *Eucalyptus* wood-associated NAC (EgWND1; Zhong et al., 2010a) are functional orthologs of the *Arabidopsis* secondary wall NACs including SND1, NST1/2 and VND6/7 (Zhong and Ye, 2007). The overexpression of these TFs in *Arabidopsis* increased the expression of secondary wall biosynthetic genes and caused concomitant ectopic deposition of lignin in secondary walls, which suggests that these TFs play a role in the control of secondary wall biosynthesis. The NAC protein subfamily, including VND, NST, SMB, and BRN of *Arabidopsis* is termed the VNS family (Ohtani et al., 2011; Xu et al., 2014). The number of VNS genes varies between plant species and does not appear to correlate with genome size or the presence of woody tissues (Zhu et al., 2012; Nakano et al., 2015). In woody angiosperms, *P. trichocarpa* has 16 (Zhong et al., 2010b; Ohtani et al., 2011; Li et al., 2012) and *E. grandis* has six VNS genes (Myburg et al., 2014; Hussey et al., 2015). In conifers, *P. abies* has four VNS genes (Nystedt et al., 2013), *P. glauca* has two VNS genes (Duval et al., 2014), and *P. pinaster* has three VNS genes (Pascual et al., 2015). Recent studies have demonstrated that the xylem transcriptomes of vascular plants are highly conserved (Li et al., 2010) and that this VNS family is conserved across a wide range of plant species, including non-vascular land plant such as bryophytes. The moss *Physcomitrella patens* contains eight VNS genes (Zhu et al., 2012; Xu et al., 2014).

Previous work in *Arabidopsis* demonstrated the existence of a complex transcriptional network involved in secondary cell wall biosynthesis (Zhong and Ye, 2007; Zhong et al., 2007, 2008, 2010a). The MYB regulators, *MYB46* and *MYB83*, are downstream of the NACs (SND1, NST1, and VND6/7) in *Arabidopsis*. The transcription factors R2R3-MYB are key in this transcriptional network, and *MYB46* is a key regulator of the biosynthesis of all three major secondary cell wall components, including cellulose, hemicellulose, and lignin (Zhong et al., 2007). They are targets of SND1 and NST1 (McCarthy et al., 2009) and situated in the second step of the transcriptional cascade. Overexpression of MYB46 or its homolog MYB83 increased the expression of other transcription factors, including MYB20, MYB42, MYB43, and MYB85, which are directly involved in the synthesis of secondary cell wall components (Zhong et al., 2008). Other transcription factors, including NACs, MYBs, and KNATs, downstream of the NAC and MYB master regulators form a complex regulatory network in secondary cell wall biosynthesis. A similar transcriptional network was identified in poplar (Zhong et al., 2010b, 2011, 2013; Lin et al., 2013), a woody angiosperm that is phylogenetically close to *Arabidopsis*. These different studies support the hypothesis that the transcriptional regulatory network governing secondary cell wall biosynthesis is largely conserved in plant species.

The transcriptional network in conifers that regulates the secondary cell wall synthesis has not been studied in detail. Bomal et al. (2008) revealed a possible relationship between MYB-like transcription factors in *P. glauca*, which is similar to the network proposed by Zhong and Ye (2007) and Zhong et al. (2007). Two NAC genes were recently identified in *P. glauca* that may act as upstream regulators of MYBs (Duval et al., 2014). However, the complexity of the regulatory gene networks controlling wood formation in *Populus* indicates that intensive research is necessary to fully define the transcriptional regulatory hierarchy in conifers.

## Conclusion

This review updates recent research advances regarding the biosynthesis and the metabolic fate of Phe in conifers. Two alternative routes have been identified: the ancient phenylpyruvate pathway that is present in many microorganisms, and the arogenate pathway that possibly evolved later during plant evolution. Besides, an efficient nitrogen recycling mechanism is required to maintain sustained growth during xylem formation. Further research efforts are necessary to clearly determine how these two pathways are regulated during vegetative development and in response to environmental changes. In regard to Phe utilization, the genetic manipulation of genes encoding key enzymes in lignin biosynthesis leads to new knowledge of considerable interest for the biochemical engineering of conifers and the generation of improved and novel forest-derived products. Progress has been made in transcriptional regulation but further research is required to elucidate the complex regulatory networks involved in the control of lignin production during wood formation. Furthermore, the occurrence of a variety of additional Phe-demanding pathways in conifers makes these plants attractive candidates for more thorough analyses aimed to clarify the metabolic utilization of this important amino acid.

## Author Contributions

MP, JE-A, FT, RC, CA, and FC contributed to writing the manuscript. RC and FT drew the figures. CA and FC conceived the review and edited the manuscript. All authors had read and approved the final manuscript.

## Conflict of Interest Statement

The authors declare that the research was conducted in the absence of any commercial or financial relationships that could be construed as a potential conflict of interest.
